# Nucleation and growth for magnesia inclusion in Fe–O–Mg melt

**DOI:** 10.1039/c8ra07728b

**Published:** 2018-11-14

**Authors:** Yuanyou Xiao, Hong Lei, Bin Yang, Guocheng Wang, Qi Wang, Wei Jin

**Affiliations:** Key Laboratory of Electromagnetic Processing of Materials, Ministry of Education, Northeastern University Shenyang Liaoning Province 110819 P. R. China leihong@epm.neu.edu.cn; School of Materials and Metallurgy, Northeastern University Shenyang Liaoning Province 110819 P. R. China; Key Laboratory of Chemical Metallurgy Engineering Liaoning Province, University of Science and Technology Liaoning Anshan Liaoning Province 114051 P. R China; Key Laboratory of Synthetic and Biological Colloids, Ministry of Education, School of Chemical and Material Engineering, Jiangnan University Wuxi Jiangsu Province 214122 P. R. China

## Abstract

The crystallization process of magnesia in iron melt begins with nucleation, which determines the structure and size of magnesia inclusions. Thus, it is necessary to have a deep insight into the crystallization of magnesia by two-step nucleation mechanisms. In this work, the two-step nucleation method was used to investigate the behavior during the early stages of magnesia inclusions crystallization. A first principles method was applied to calculate the thermodynamic properties of magnesia crystal from various cluster structures for the formation of magnesia inclusions. Based on the numerical results, the nucleation mechanism of magnesia in liquid iron has been discussed. The magnesia clusters appear as the structural units for Mg-deoxidation reaction in the liquid iron, and the residual magnesia clusters are the reason for the supersaturation ratio or the excess oxygen for MgO formation in the liquid iron. Based on the comparison between Mg-deoxidation equilibrium experiments and numerical results, the previous experiments may be in a different thermodynamic state. The equilibrium reaction product should be not only magnesia clusters but also bulk-magnesia in those equilibrium experiments.

## Abstract 2

The crystallization process of magnesia involves two steps.

## Graphical abstract

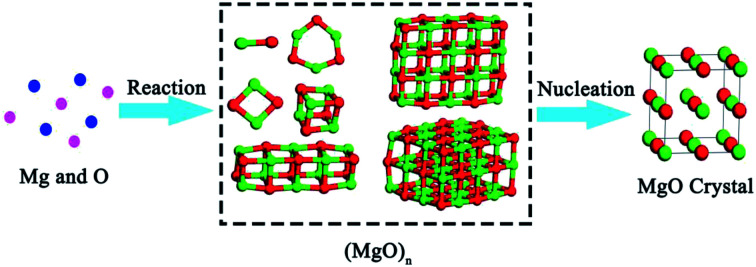

## 1. Introduction

Non-metallic inclusions are one of the key factors to affect the quality of steel products because their properties differ to those of the steel matrix, and they act as stress raisers and crack sources. Lowe and Mitchell^1^ suggested that nonmetallic inclusions are of almost no hazard to the mechanical properties of steel if the size of the inclusion particle is less than 1 μm and the distance between two particles is greater than 10 μm in the steel matrix. In addition, fine inclusions can be utilized as nucleation sites for phase transformation and play a positive role on the nucleation of acicular ferrite.^2–4^ Consequently, the control of inclusions size can be one of the effective measures to improve steel performance. Therefore, it is necessary to have a deep insight into the crystallization of inclusions in the iron melt.

Magnesium is one of the most important deoxidizers in the steel-making process and has received great interest and attention for its strong affinity to oxygen in the iron melt. The Mg-deoxidation in iron melt has been investigated for many years.^5–15^ Most of the researchers focused on the thermodynamic equilibrium relationship between the dissolved Mg and O^5–8^ or the Mg-deoxidation experiment.^9–14^ The magnesium deoxidation reaction and its standard Gibbs free energy change can be written as:^15^

1 [Mg] + [O] = MgO(s) Δ*G*^θ^_MgO(s)_ = −728418 + 238.338*T* (J mol^−1^)

However, such an equation only reveals the thermodynamic properties of the formation of MgO, and does not provide a clear picture about the structure of intermediate phase or the nucleation pathway leading from the dissolved state to the solid crystal. The early stages of the crystallization of MgO inclusions play a important role in determining the structure and size of MgO inclusions in the iron melt. Thus, the lack of knowledge about the formation pathways of critical nucleus of magnesia inclusions at atom scale hinders the control for the physical and chemical properties of magnesia inclusions.

The two-step nucleation method (TSNM), has been successfully applied to the solutions, and has provided a new method to investigate the mechanism of metastable structure growth and nuclei formation.^16–23^ Wasai *et al.*^24^ found the metastable alumina and silica in Al-deoxidation experiments by the ultra-rapid cooling method. This means that the nucleation of inclusions in molten steel contains an intermediate process. Based on the TSNM, Zong *et al.*^25^ investigated the behavior during the early stages of MgO·Al_2_O_3_ spinel inclusion crystallizations in steels and obtained the structure and thermodynamic property of intermediate products (MgO)_*n*_ and (Al_2_O_3_)_*n*_ clusters. Zong *et al.*^25^ reported that the nucleation pathway which derives from a variety of metastable structures in earlier crystal nucleation stages was stronger than the classical pathway. Wang *et al.*^26–28^ also suggested that the formation of inclusions in molten steel follows a two-step nucleation mechanism. Firstly, the deoxidizers react with the dissolved oxygen in molten steel to form various intermediate phase, and then the intermediate phase transform into the stable crystal.

The thermodynamics about intermediate phase of magnesia (MgO)_*n*_ is useful to reveal the mechanism on Mg-deoxidation nucleation in liquid iron. Understanding the thermodynamics of (MgO)_*n*_ clusters forming in iron melt is important to explore the relationship between the size of magnesia inclusion and Mg-deoxidation reaction. The (MgO)_*n*_ clusters have been reported by many researchers.^25,29–34^ Chen *et al.*^29^ studied the structures and stabilities of (MgO)_*n*_ (*n* = 2–40) nanoclusters. Dong *et al.*^32^ provided the structures of (MgO)_3*n*_ (2 ≤ *n* ≤ 10) clusters. However, most of them focused on the atomic structures and electronic properties of (MgO)_*n*_ clusters, while few of them provided the thermodynamic properties (MgO)_*n*_ clusters. There are few references about the nucleation of magnesia in molten steel by TSNM.

In this work, TSNM is used to investigate the behavior during the early stages of magnesia inclusions crystallization. Numerical simulation is carried out to study the structure and thermodynamic property of metastable phase of magnesia before critical nucleus by first principle method. Then, we investigate the nucleation mechanism of magnesia in the liquid iron. Base on the comparison between the experimental data and the numerical results, we discuss the relationship between the size of magnesia inclusions and Mg-deoxidation reaction.

## 2. Theoretical calculations

### 2.1. Calculation details

The geometry optimization and the thermodynamic calculation for (MgO)_*n*_ cluster and MgO crystal were carried out by Dmol3 module of Materials Studio 7.0, which is a molecular orbital theory computational program based on density functional theory. The framework of the generalized gradient approximation GGA proposed by Perdew, Burke, and Ernzerhof^9^ was used during the calculations. The thermodynamic properties of (MgO)_*n*_ cluster and MgO crystal were obtained by the vibrational analysis or Hessian evaluation as the functions of the temperature. The entropy *S* and enthalpy *H* of (MgO)_*n*_ cluster and MgO crystal are calculated as^35^2
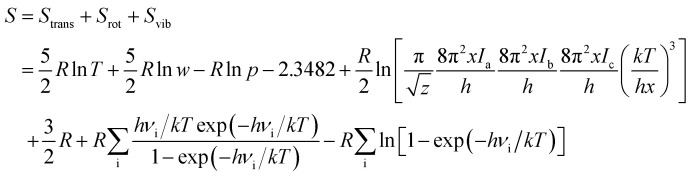
3
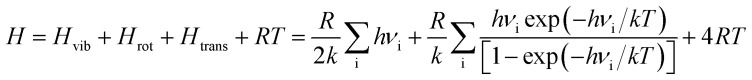
where *H*_trans_ and *S*_trans_ are the enthalpy and the entropy of translation, respectively. *H*_rot_ and *S*_rot_ are the enthalpy and the entropy of rotation, respectively. *H*_vib_ and *S*_vib_ are the enthalpy and the entropy of vibration, respectively. *w* is the molecular mass; *h* is Planck's constant; *k* is the Boltzmann constant; *R* is the ideal gas constant; *ν*_i_ is the vibrational frequency. *T* is the absolute temperature; *p* is the pressure; *z* is the symmetry number; *x* is the molar concentration of the molecules; and *I*_a(b,c)_ is the moment of inertia. The Gibbs free energy of (MgO)_*n*_ cluster and MgO crystal is calculated by *G* = *E* (0 K) + *H* − *TS*, where *E* (0 K) is the total energy at 0 K. In addition, the heat capacity at constant pressure *C*_P_ is computed as^36^4
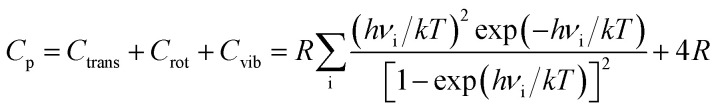
*C*_trans_, *C*_rot_ and *C*_vib_ are the heat capacities of translation, rotation and vibration, respectively.

### 2.2. Structures and thermodynamic properties

**Fig. 1:**
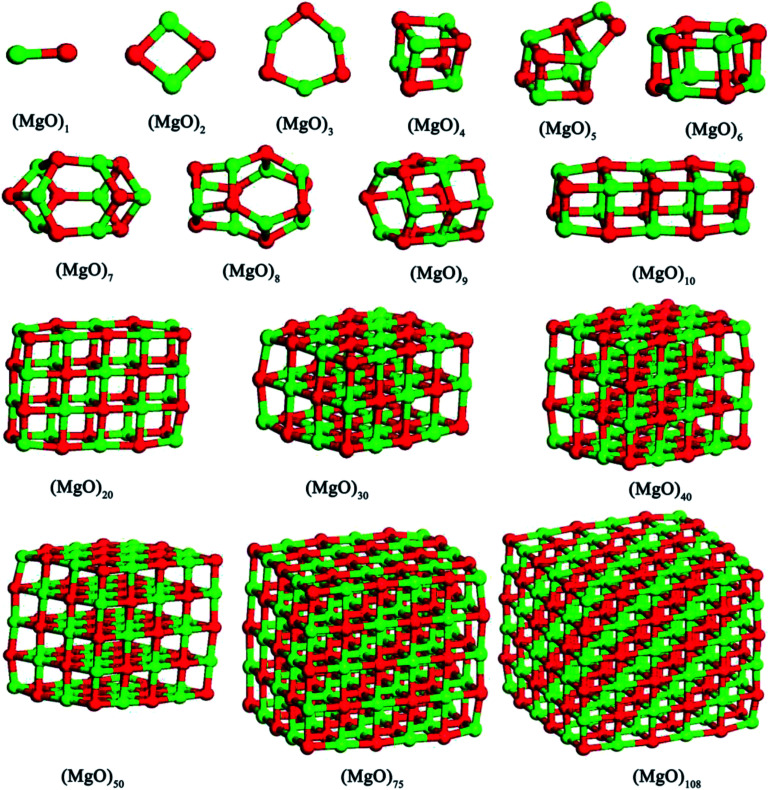
Stable structures of magnesia clusters (MgO)_*n*_ (*n* = 1–108) cluster.

**Fig. 2:**
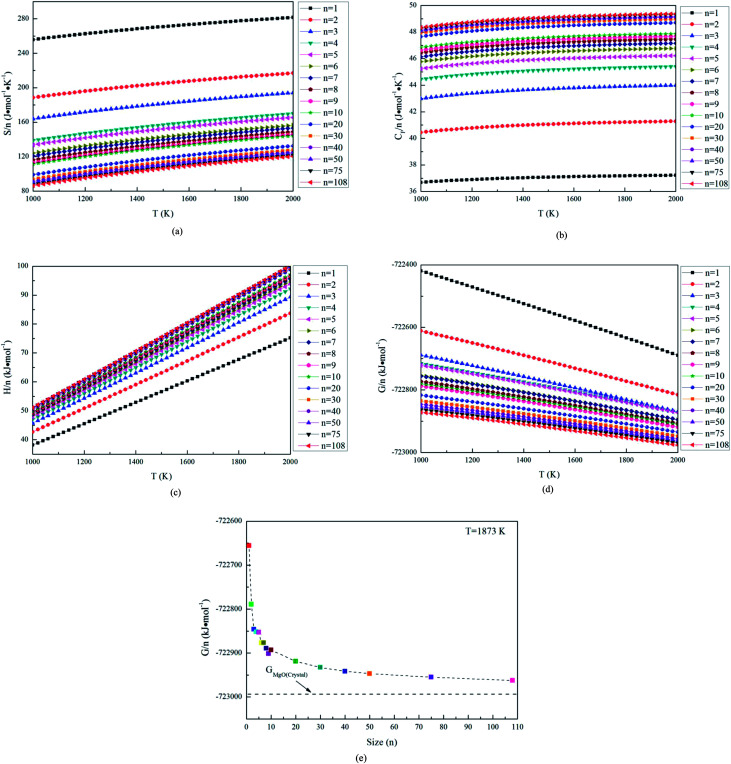
Thermodynamics properties of magnesia clusters, (a) *S*/*n*, (b) *C*_P_/*n*, (c) *H*/*n*, (d) *G*/*n*, (e) *G*/*n* at 1873 K.

In this studies, the possible initial structures of (MgO)_*n*_ (*n* = 2–30) cluster were selected from the lowest energy structures in the previous studies.^24,29–31^ The clusters of (MgO)_2_ and (MgO)_3_ are planar and ring-like structure, while the clusters of (MgO)_*n*_ (*n* > 10) are cuboid structure. It should be noted that the most stable clusters of (MgO)_*n*_ (*n* = 10–30) are similar to the fragment of bulk magnesia crystal. Therefore, the initial structures of (MgO)_*n*_ (*n* = 40, 50, 75 and 108) clusters were selected from the fragments of the bulk magnesia crystal. The stable structures of (MgO)_*n*_ (*n* = 1–108) clusters are shown in Fig. 1. The average distance of Mg–O bond increases with the increasing size and is slowly close to the value of MgO crystal (2.106 Å). Table 1 gives the surface area and volume of (MgO)_*n*_ (*n* = 4–108) cluster. Both the total average energy of clusters at 0 K E (0 K)/*n* and the average binding energy of clusters *E*_b_ at 1873 K decrease with the increasing size. This result shows that the stability of magnesia cluster increases with the increasing size. Fig. 2 shows the thermodynamic properties of (MgO)_*n*_ (*n* = 1–108) clusters within the temperature range from 1000 K to 2000 K. *H*/*n*, *S*/*n* and *C*_P_/*n* increase with the increasing temperature. *H*/*n* and *C*_P_/*n* increase with the increasing size, while *S*/*n* decreases with the increasing size. In addition, *G*/*n* decreases with the increasing temperature and size, and the Gibbs free energy of crystals *G*_MgO(crystal)_ is less than *G*/*n*. This result shows that the crystals is more stable than the clusters, and the stability of magnesia cluster increases with the increasing size. And the Gibbs free energy of clusters gets closer to that of crystals gradually with the increasing size. Therefore, the stability of magnesia cluster is gradually close to that of MgO crystal, and the clusters tend to grow up and nucleate.

**Table 1:** Energies and structure properties of magnesia clusters (MgO)_*n*_ (*n* = 1–108) cluster

Size (*n*)	*E*_b_ (kJ mol^−1^)	*E* (0 K), (kJ mol^−1^)	Surface area (m^2^)	Volume (m^3^)	Symmetry
*n* = 1	−307.364	−722201.465	—	—	C1
*n* = 2	−571.349	−722465.450	—	—	C1
*n* = 3	−676.207	−722570.308	—	—	C1
*n* = 4	−729.869	−722623.969	—	—	C1
*n* = 5	−741.136	−722635.237	2.333 × 10^−19^	7.669 × 10^−30^	C1
*n* = 6	−783.711	−722677.811	3.046 × 10^−19^	1.150 × 10^−29^	C1
*n* = 7	−790.051	−722684.151	4.293 × 10^−19^	1.513 × 10^−29^	C1
*n* = 8	−811.915	−722706.015	5.134 × 10^−19^	3.144 × 10^−29^	C1
*n* = 9	−830.057	−722724.158	5.822 × 10^−19^	2.231 × 10^−29^	C1
*n* = 10	−824.672	−722718.772	6.313 × 10^−19^	1.772 × 10^−29^	C1
*n* = 20	−857.671	−722768.543	6.914 × 10^−19^	3.008 × 10^−29^	C1
*n* = 30	−874.442	−722791.969	1.481 × 10^−18^	9.092 × 10^−29^	C1
*n* = 40	−884.685	−722805.852	2.012 × 10^−18^	1.819 × 10^−29^	C1
*n* = 50	−897.869	−722813.769	2.626 × 10^−18^	2.877 × 10^−29^	C1
*n* = 75	−911.751	−722826.206	3.241 × 10^−18^	3.907 × 10^−29^	C1
*n* = 108	−919.668	−722836.086	4.584 × 10^−18^	6.629 × 10^−29^	C1

## 3. Discussions

### 3.1. Nucleation and Gibbs energy changes in Fe–O–Mg melt

**Fig. 3:**
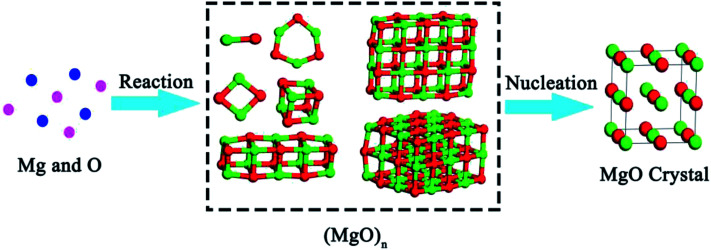
Multi-step nucleation pathway of magnesia crystal.

According to the TSNM, the crystallization process of magnesia involves in two steps. As shown in Fig. 3, [Mg] reacts with [O] to form various magnesia cluster structures in an Fe–O–Mg melt at first. This process can be expressed as

5 [Mg] + [O] = 1/*n*(MgO)_*n*_

In the second step, the magnesia clusters can transform into a crystal. Such a process can be expressed by

6 1/*n*(MgO)_*n*_ = MgO (crystal)

The Gibbs free energy changes of eqn (5) Δ*G*^θ^_*n*_ can be calculated as

**Fig. 4:**
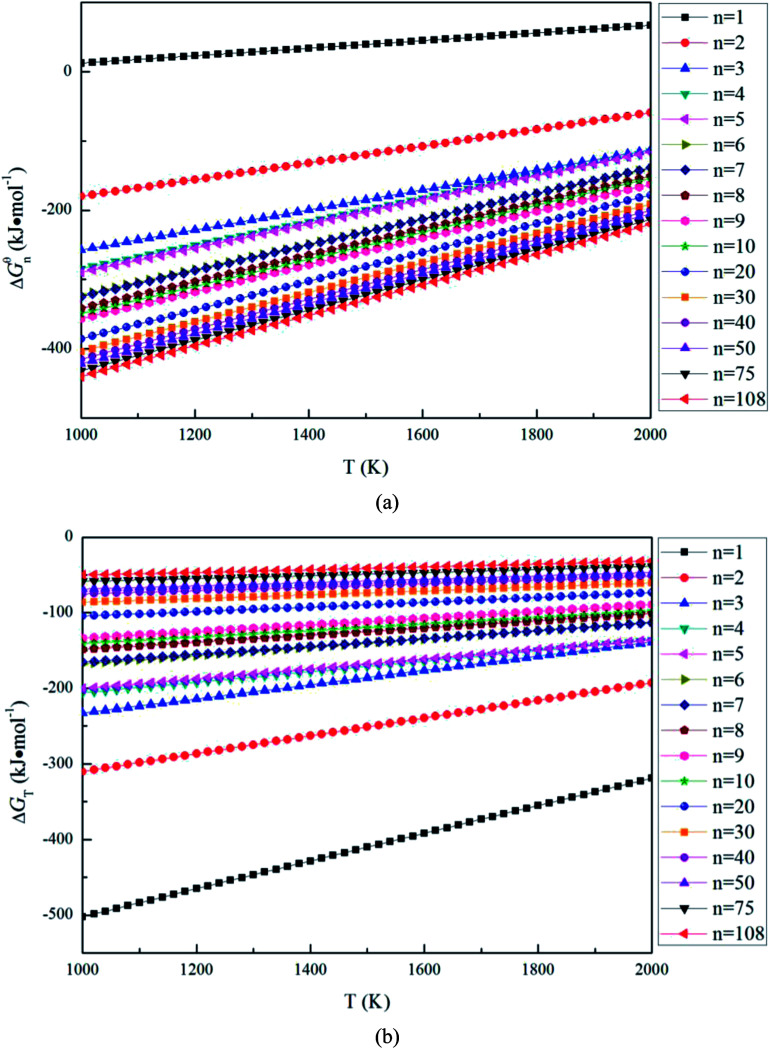
Gibbs free energy change for the magnesia clusters (MgO)_*n*_ (*n* = 1–108) formation reaction, (a) Gibbs free energy changes for eqn (5), (b) Gibbs free energy changes for eqn (6).

7 Δ*G*^θ^_*n*_ = Δ*G*^θ^_MgO(s)_ − [*G*_MgO(crystal)_ − (1/*n*)*G*_(MgO)_*n*__]

where Δ*G*^θ^_MgO(s)_ is the Gibbs free energy change for eqn (1), (1/*n*)*G*_(MgO)_*n*__ is the Gibbs free energy of 1/*n* (MgO)_*n*_. Fig. 4(a) shows that the Gibbs free energy changes Δ*G*^θ^_*n*_ for the magnesia clusters (MgO)_*n*_ (*n* = 1–108) formation reaction decreases with the decrease of the temperature (1000 to 2000 K) and the size. Such a result indicates that the thermodynamic driving forces for the formation of (MgO)_*n*_ (*n* = 1–108) increases with the decrease of the temperature and the size. The value of Δ*G*^θ^_*n*_ for (MgO)_1_ is not negative, this means the (MgO)_1_ is not stable at the temperature range from 1000 to 2000 K.

The Gibbs free energy changes for eqn (6) Δ*G*_T_ can be calculated as

8 Δ*G*_T_ = *G*_MgO(crystal)_ − (1/*n*)*G*_(MgO)_*n*__

where *n* is the number of units in a cluster. Fig. 4(b) shows the Gibbs free energy changes Δ*G*_T_ for magnesia clusters (MgO)_*n*_ (*n* = 1–108) to transform into magnesia crystal. The values of Δ*G*_T_ are negative, and decrease with the decreasing size. This means the thermodynamic driving force for magnesia clusters to transform into magnesia crystal increases with the decreasing size.

The Gibbs free energy change (Δ*G*) of one mole of the liquid Fe–O–Mg system, when *n*_0_ nuclei with radius *r* are formed, is expressed as:^37^

9 Δ*G* = Δ*G*_R_ + Δ*G*_I_ + Δ*G*_L_ = *n*_0_(Δ*g*_R_ + Δ*g*_I_ + Δ*g*_L_)

where Δ*G*_R_ is the Gibbs free energy change for magnesia formation reaction, Δ*G*_I_ is the interface free energy change of magnesia formation, Δ*G*_L_ is the Gibbs free energy change of parent liquid iron before and after nucleation, *n*_0_ is the number of nuclei in one mole of the liquid Fe–O–Mg system.

The interfacial free energy between magnesia and liquid iron is calculated as

10 Δ*G*_I_ = *Aσ*

where *A* is the surface area of magnesia, and *σ* can be written as^38,39^

11 *σ* = 0.918 − 0.033 ln(1 + 130[%O]) (N m^−1^)

Δ*G*_L_ is written as12
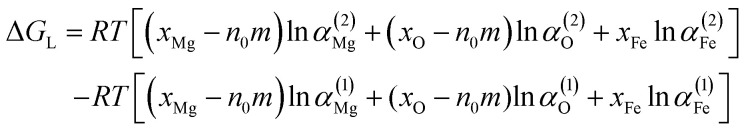
where *α*_i_ is the activity of i, the superscripts (1) and (2) are the parent iron phases before nucleation and after nucleation, and *x*_i_ is the initial molar fraction of i. Wasai *et al.*^30^ reported that the Gibbs free energy change of parent liquid iron before and after nucleation are almost zero in the small-radius region. Therefore, Δ*G*_L_ can be neglected in this work. Δ*G*_R_ is written as

13 Δ*G*_R_ = *B*Δ*G*^θ^_*n*_

where *B* is equal to *V*/*V*_m_, *V* is volume of magnesia, *V*_m_ is the molar volume of magnesia clusters. The clusters (MgO)_*n*_ (*n* = 1–3), which are not three dimensional structure, were not included in this section.

**Fig. 5:**
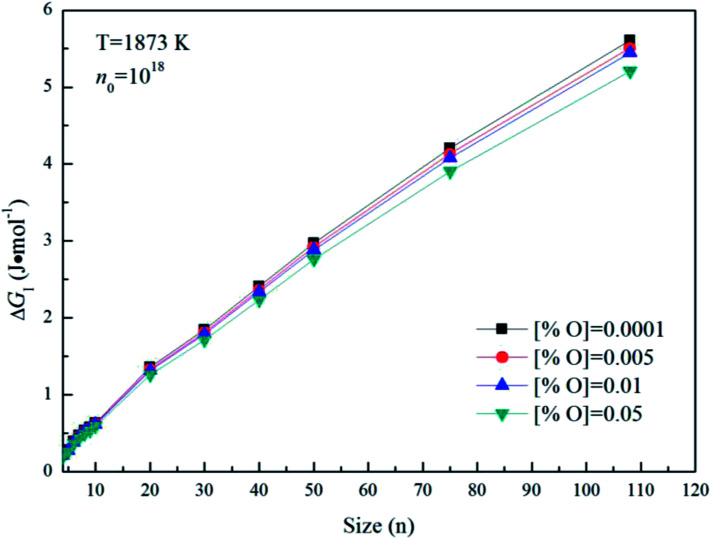
The interfacial free energy between magnesia clusters (MgO)_*n*_ (*n* = 4–108) and liquid iron for various initial oxygen contents (*n*_0_ = 10^18^).

**Fig. 6:**
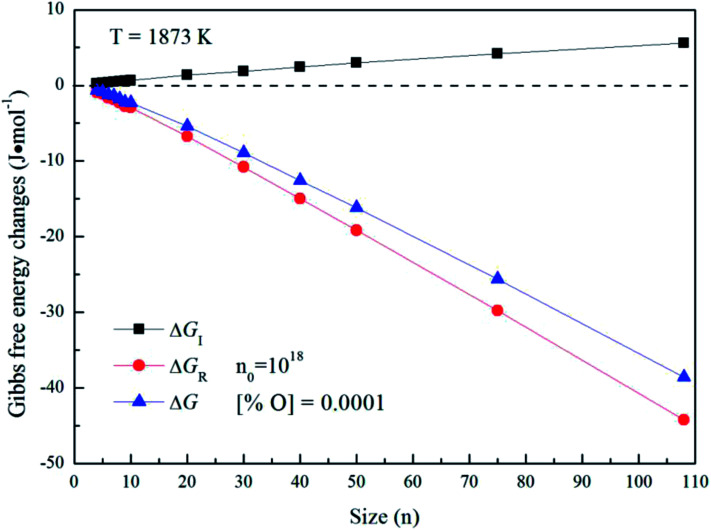
Gibbs free energy changes of Δ*G*_I_, Δ*G*_R_ and Δ*G* for initial oxygen contents of [% O] = 0.0001.

Fig. 5 gives the interfacial free energy between magnesia clusters (MgO)_*n*_ (*n* = 4–108) and liquid iron in case of various initial oxygen contents. The value of Δ*G*_I_ is positive and increases as *r* increases. This result indicates that energy barrier for the formation of magnesia clusters in liquid iron increases with the increasing size. Moreover, the changes of initial oxygen contents have little effect on the interfacial free energies. Fig. 6 shows the Gibbs free energy changes of Δ*G*_I_, Δ*G*_R_ and Δ*G* in the case of initial oxygen contents [% O] = 0.0001. The value of Δ*G* is negative, and almost equals the value of Δ*G*_R_. This result indicates that the interfacial free energy has little effect on Δ*G*. In other words, the magnesia clusters can form spontaneously by overcoming a low-energy barrier.

### 3.2. Growth of magnesia clusters and excess oxygen in Fe–O–Mg melt

**Fig. 7:**
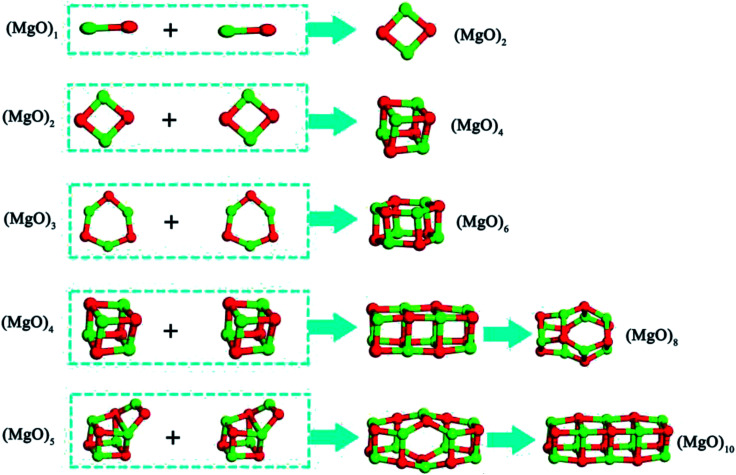
Aggregation reaction of magnesia clusters.

The small clusters can grow up by the aggregation among two or more clusters.^40–45^ The smaller magnesia clusters are deposited on their nearest magnesia cluster, which may provide a further way to directly assemble or grow up. The magnesia clusters are more reactive than their atoms in the bulk magnesia crystal because of the larger exposed surfaces and the higher surface reactivity. Thus, it is easily for the magnesia clusters to adsorb and aggregate with each other compared with the magnesia crystals. Such a fact leads to the formation of nuclei that can act as the centers of crystallization. As shown in Fig. 7, two same clusters (MgO)_*n*_ (*n* = 1–3) can aggregate into the most stable clusters (MgO)_2_, (MgO)_4_ and (MgO)_6_ directly, while the two same clusters (MgO)_*n*_ (*n* = 4–5) aggregate into the most stable structure need through a intermediates structure.

The aggregation reactions between the (MgO)_*n*_ and (MgO)_*m*_ are expressed as

14 (MgO)_*n*_ + (MgO)_*m*_ = (MgO)_*n*+*m*_

The Gibbs free energy changes for eqn (14) Δ*G*_*n*+*m*_ can be written as

**Fig. 8:**
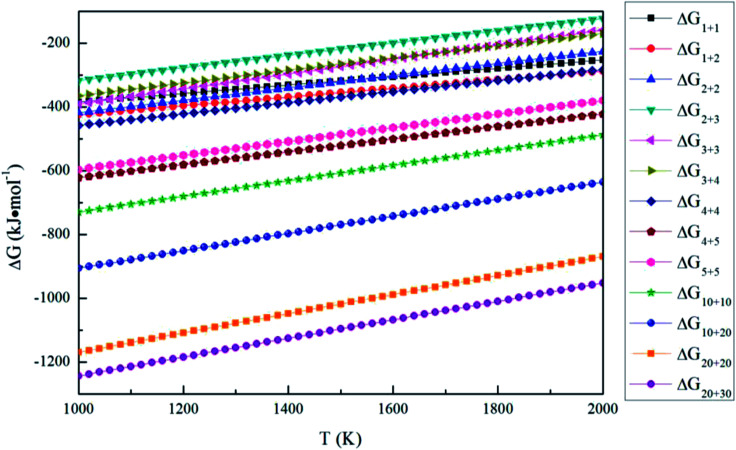
Gibbs free energy changes for the aggregation reaction of magnesia clusters.

15 Δ*G*_*n*+*m*_ = *G*_*n*+*m*_ − *G*_*n*_ − *G*_*m*_

where *G*_*n*+*m*_, *G*_*n*_ and *G*_*n*_ are the Gibbs free energy of (MgO)_*n*+*m*_, (MgO)_*n*_ and (MgO)_*m*_, respectively. Fig. 8 shows the Gibbs free energy changes for aggregation reactions between (MgO)_*n*_ and (MgO)_*m*_ (*n*, *m* = 1–30). The Gibbs free energy changes for aggregation reactions are negative in the temperature range of 1000–2000 K, so the aggregation reactions between (MgO)_*n*_ and (MgO)_*m*_ (*n*, *m* = 1–30) can also occur spontaneously. All the Δ*G*_*n*+*m*_ (*n*, *m* = 1–30) of aggregation reactions decrease as the temperature decreases. Such a result indicates that it is easier for the aggregation reactions to occur at lower temperatures.

However, as the Mg-deoxidation reaction proceeds, the thermodynamic driving force decreased gradually with the decreasing supersaturation ratio in Mg-deoxidation process. The supersaturation ratio *S* for the formation of solid magnesia in Mg-deoxidation process can be written as^46^16
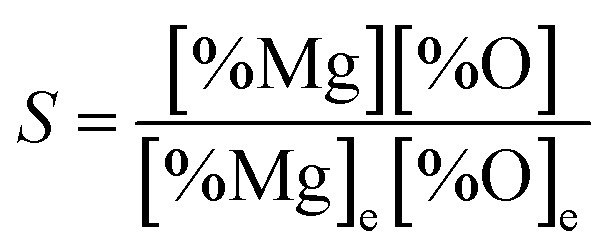
where [% Mg] and [% O] are the experimental value; [% Mg]_e_ and [% O]_e_ are the equilibrium values. Suito *et al.*^38^ reported that the calculated and experimental values for the logarithm of the critical degree of supersaturation (log S*) are 2.447 and 0.924, respectively. Because of the high-energy barrier, it is very difficult for the magnesia clusters to grow up into the final bulk magnesia or to decompose into Mg and O at the later deoxidation period. Moreover, the collision probability is low and the magnesia clusters are not large enough to float upward. As a result, the magnesia clusters can appear as the structural units in Mg-deoxidation reaction in the liquid iron, and may remain as suspending inclusions in the liquid iron for a long time. The oxygen content that exceeds the equilibrium value is called as the excess oxygen and the excess oxygen should be in the supersaturated state.^37^ Wasai and Mukai^37^ suggested the suspension of fine inclusions is a likely cause of excess oxygen. Therefore, the behavior of the residual magnesia clusters may be the reason for the supersaturation ratio or the excess oxygen for magnesia formation in liquid iron. These magnesia clusters, which may be called as the excess oxygen, cannot transform into bulk-magnesia at the steel-making temperature.

### 3.3. Mg-deoxidation equilibrium in liquid iron

**Fig. 9:**
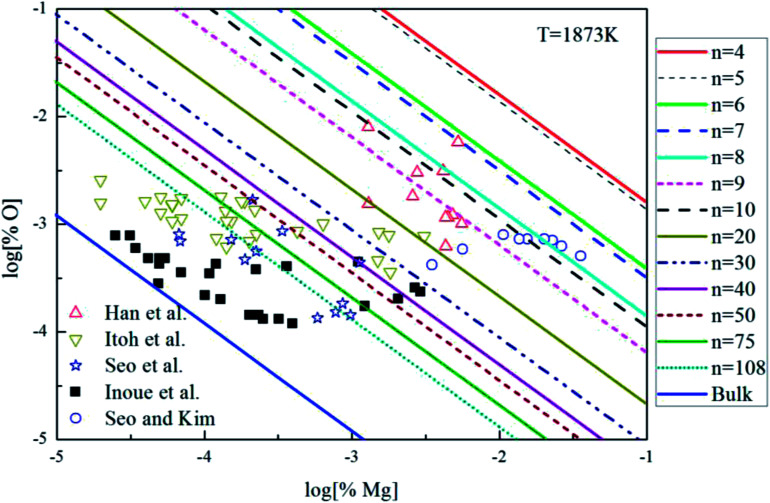
Equilibrium curves of magnesia equilibrated in liquid iron at 1873 K.

The Mg-deoxidation equilibrium in liquid iron has been investigated by many researchers.^10–14^ Seo and Kim^10^ and Han *et al.*^12^ held their Mg-deoxidation experiments in a closed magnesia crucible in the case of several hundred parts per million oxygen by using Mg vapor. Itoh *et al.*^11^ performed the similar experiments in open dolomite crucibles under a mixture atmosphere of argon and hydrogen in a high frequency induction furnace. Seo *et al.*^14^ held their experiments in a specially designed high frequency induction furnace with a strong agitation of melt by adding Ni–Mg alloys. Fig. 9 shows that the equilibrium concentration of dissolved magnesium is fluctuating within the range of 1.5 × 10^−5^ < [% Mg] < 0.032. Moreover, the equilibrium concentration of dissolved oxygen decreases with the increasing [% Mg] if the equilibrium concentration [% Mg] < 0.001, but the equilibrium concentration of dissolved oxygen increases with the increasing [% Mg] if the equilibrium concentration [% Mg] > 0.001. It should be noted that the difference among the equilibrium concentration of dissolved oxygen is more than one order of magnitude in the case of the same concentration of dissolved magnesium. Jung *et al.*^47^ suggested that the distribution of the dissolved magnesium and oxygen atoms could not be independent and random, but these dissolved magnesia and oxygen atoms had a strong tendency to form dissolved associated compound Mg–O *etc*, which is a kind of metastable phase in the liquid iron. The present authors suggested that the previous experimental data are obtained in the different thermodynamic states which depend on the different experimental conditions. The thermodynamics of Mg-deoxidation reaction in liquid iron has a close relationship with that of metastable phase, such as dissolved associated compound Mg–O, (MgO)_*n*_ clusters *etc.*

Fig. 9 shows the thermodynamic curves of magnesia clusters (MgO)_*n*_ (*n* = 4–108) in equilibrium with liquid iron during Mg-deoxidation process at 1873 K in present work. All the experimental data are covered by the region between the magnesia clusters equilibrium curves (MgO)_*n*_ (*n* = 4) and the bulk-magnesia equilibrium curve. This fact suggests that these experiments are in different thermodynamic state. In other words, [Mg] and [O] in equilibrium state depend not only on bulk-magnesia inclusion but also on various size magnesia clusters. It suggests that the equilibrium reaction product should be not only magnesia clusters but also bulk-magnesia in those equilibrium experiments. In addition, the magnesia clusters equilibrium curves are close to the bulk-magnesia equilibrium curve gradually with the increasing size of magnesia inclusion. Therefore, most of the Mg-deoxidation reaction experiments do not reach the final equilibrium but gradually approach the final equilibrium in different degree.

## 4. Conclusions

(1) The Gibbs free energies are negative for the formation, aggregation and transformation of magnesia cluster.

(2) The magnesia clusters appear as the structural units in Mg-deoxidation reaction for liquid iron. The residual metastable magnesia is the reason for the supersaturation ratio or the excess oxygen for MgO formation in liquid iron.

(3) The previous experimental data is obtained in the different thermodynamic state. And the difference among the experiments data comes from the size effect of MgO clusters.

## Conflicts of interest

There are no conflicts to declare.

## Supplementary Material
